# Targeting on Gut Microbiota-Derived Metabolite Trimethylamine to Protect Adult Male Rat Offspring against Hypertension Programmed by Combined Maternal High-Fructose Intake and Dioxin Exposure

**DOI:** 10.3390/ijms21155488

**Published:** 2020-07-31

**Authors:** Chien-Ning Hsu, Julie Y. H. Chan, Hong-Ren Yu, Wei-Chia Lee, Kay L. H. Wu, Guo-Ping Chang-Chien, Sufan Lin, Chih-Yao Hou, You-Lin Tain

**Affiliations:** 1 Department of Pharmacy, Kaohsiung Chang Gung Memorial Hospital, Kaohsiung 833, Taiwan; cnhsu@cgmh.org.tw; 2 School of Pharmacy, Kaohsiung Medical University, Kaohsiung 807, Taiwan; 3 Institute for Translational Research in Biomedicine, Kaohsiung Chang Gung Memorial Hospital and Chang Gung University College of Medicine, Kaohsiung 833, Taiwan; jchan@cgmh.org.tw (J.Y.H.C.); wlh0701@yahoo.com.tw (K.L.H.W.); 4 Department of Pediatrics, Kaohsiung Chang Gung Memorial Hospital and Chang Gung University College of Medicine, Kaohsiung 833, Taiwan; yuu2002@cgmh.org.tw; 5 Department of Urology, Kaohsiung Chang Gung Memorial Hospital and Chang Gung University College of Medicine, Kaohsiung 833, Taiwan; dinor666@ms32.hinet.net; 6 Center for Environmental Toxin and Emerging-Contaminant Research, Cheng Shiu University, Kaohsiung 833, Taiwan; guoping@csu.edu.tw (G.-P.C.-C.); linsufan2003@gmail.com (S.L.); 7 Super Micro Mass Research and Technology Center, Cheng Shiu University, Kaohsiung 833, Taiwan; 8 Department of Seafood Science, National Kaohsiung University of Science and Technology, Kaohsiung 811, Taiwan; chihyaohou@webmail.nkmu.edu.tw

**Keywords:** developmental origins of health and disease (DOHaD), fructose, gut microbiota, hypertension, 2,3,7,8-tetrachlorodibenzo-p-dioxin (TCDD), trimethylamine, trimethylamine N-oxide (TMAO)

## Abstract

Gut microbiota-dependent metabolites, in particular trimethylamine (TMA), are linked to hypertension. Maternal 2,3,7,8-tetrachlorodibenzo-p-dioxin (TCDD) exposure or consumption of food high in fructose (HFR) can induce hypertension in adult offspring. We examined whether 3,3-maternal dimethyl-1-butanol (DMB, an inhibitor of TMA formation) therapy can protect adult offspring against hypertension arising from combined HFR and TCDD exposure. Pregnant Sprague–Dawley rats received regular chow or chow supplemented with fructose (60% diet by weight) throughout pregnancy and lactation. Additionally, the pregnant dams received TCDD (200 ng/kg BW orally) or a corn oil vehicle on days 14 and 21 of gestation, and days 7 and 14 after birth. Some mother rats received 1% DMB in their drinking water throughout pregnancy and lactation. Six groups of male offspring were studied (*n* = 8 for each group): regular chow (CV), high-fructose diet (HFR), regular diet+TCDD exposure (CT), HFR+TCDD exposure (HRT), high-fructose diet+DMB treatment (HRD), and HFR+TCDD+DMB treatment (HRTD). Our data showed that TCDD exacerbates HFR-induced elevation of blood pressure in male adult offspring, which was prevented by maternal DMB administration. We observed that different maternal insults induced distinct enterotypes in adult offspring. The beneficial effects of DMB are related to alterations of gut microbiota, the increase in nitric oxide (NO) bioavailability, the balance of the renin-angiotensin system, and antagonization of aryl hydrocarbon receptor (AHR) signaling. Our findings cast new light on the role of early intervention targeting of the gut microbiota-dependent metabolite TMA, which may allow us to prevent the development of hypertension programmed by maternal excessive fructose intake and environmental dioxin exposure.

## 1. Introduction

The global consumption of fructose per capita has grown exponentially during the past few decades [1]. A diet high in fructose consumed during pregnancy not only induces adverse effects for mothers, but also for their adult offspring [2,3]. The origins of many adult diseases can be traced to negative experiences early in life. This notion is termed the developmental origins of health and disease (DOHaD) [4]. Our previous study reported that offspring exposed to maternal high-fructose diets developed hypertension in adulthood [5]. Progression of normotensive children to hypertensive adults can occur synergistically with multiple adverse environmental conditions [5]. Exposure to environmental chemicals, such as dioxins, can increase the risk of hypertension [6]. Specifically, maternal 2,3,7,8-tetrachlorodibenzo-p-dioxin (TCDD) exposure has been reported to exacerbate hypertension in adult male offspring in various models of developmental programming [7,8].

Several mechanisms have been proposed as underlying the pathogenesis of hypertension and kidney disease of developmental origin [9], such as nitric oxide (NO) deficiency, gut microbiota dysbiosis, and dysregulation of the renin-angiotensin system (RAS). Gut microbiota dysbiosis has been reported to be associated with hypertension [10]. Cumulative evidence in several animal models of hypertension supports the idea that the development of hypertension is interrelated with gut microbiota dysbiosis [10,11], including the high-fructose (HFR) model [12]. Certain microbiota-generated metabolites have been found to be linked with hypertension, like trimethylamine N-oxide (TMAO) and trimethylamine (TMA) [13]. Gut bacteria metabolize dietary components such as choline and carnitine to TMA, which is subsequently oxidized to TMAO by hepatic enzyme flavin-containing monooxygenases (FMOs) [13]. Our previous study demonstrated that maternal HFR-induced hypertension in adult offspring is relevant to increases in plasma TMA [12]. Additionally, TCDD, an aryl hydrocarbon receptor (AHR) ligand, has been linked with hypertension [14]. Our objective in this study was first to determine whether TCDD exposure exacerbates HFR-induced hypertension via mediation of the gut microbiota-dependent TMA-TMAO metabolic pathway, AHR signaling, RAS, and the NO pathway.

Despite probiotics being reported to prevent HFR-induced hypertension [15], little is known about whether targeting the gut microbiota-derived metabolite TMA can prevent combined HFR and TCDD exposure-induced hypertension in adult offspring. 3,3-Dimethyl-1-butanol (DMB), a structural analog of choline, has been shown to inhibit distinct microbial TMA lyases and reduce plasma TMAO levels [16]. Additionally, we recently reported that maternal DMB treatment protected adult offspring against HFR-induced hypertension, which was associated with reduced plasma levels of TMA and TMAO in the adult offspring [12]. Therefore, the second aim of our study was to examine whether DMB (an inhibitor for TMA formation) treatment can prevent combined HFR plus TCDD exposure-induced hypertension.

## 2. Results

### 2.1. Blood Pressure and Renal Function

**Figure 1:**
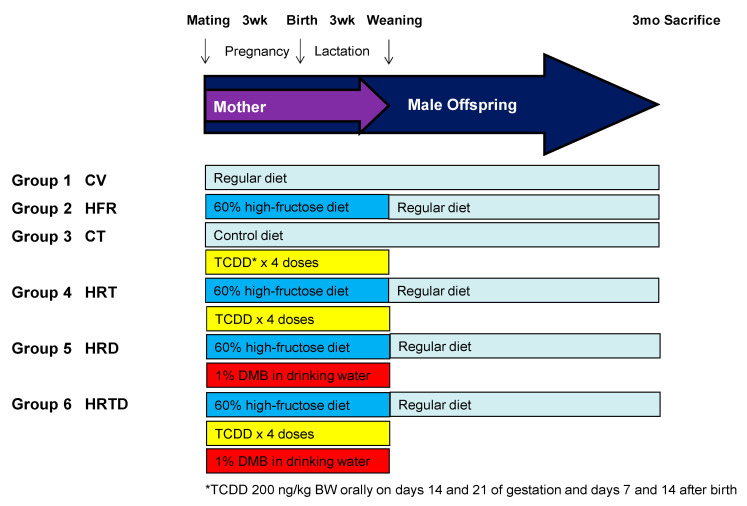
Experimental protocol used in the current study. CV = control rats received regular diet and vehicle; HFR = rats received high-fructose diet; CT = rats received regular diet and TCDD exposure; HRT = rats received high-fructose diet and TCDD exposure; HRD = rats received high-fructose diet and DMB treatment; HRDT = rats received high-fructose diet, TCDD exposure, and DMB treatment; TCDD = 2,3,7,8-tetrachlorodibenzo-p-dioxin; DMB = 3,3-Dimethyl-1-butanol.

Figure 1 illustrates the experimental protocol. We studied six groups of male offspring, including rats that received a regular diet and vehicle (CV); rats that received a high-fructose diet (HFR); rats that received a regular diet and TCDD exposure (CT); rats that received a high-fructose diet and TCDD exposure (HRT); rats that received a high-fructose diet and DMB treatment (HRD); and rats that received a high-fructose diet, TCDD+ exposure, and DMB treatment (HRTD). Table 1 shows no dead pups in any group. The body weight (BW) did not differ among most groups, except the HRD group had a higher BW than the HFR group (Table 1). Combined HFR and TCDD exposure caused a higher kidney weight than HFR alone, while the kidney weight-to-BW ratio was comparable.

**Figure 2:**
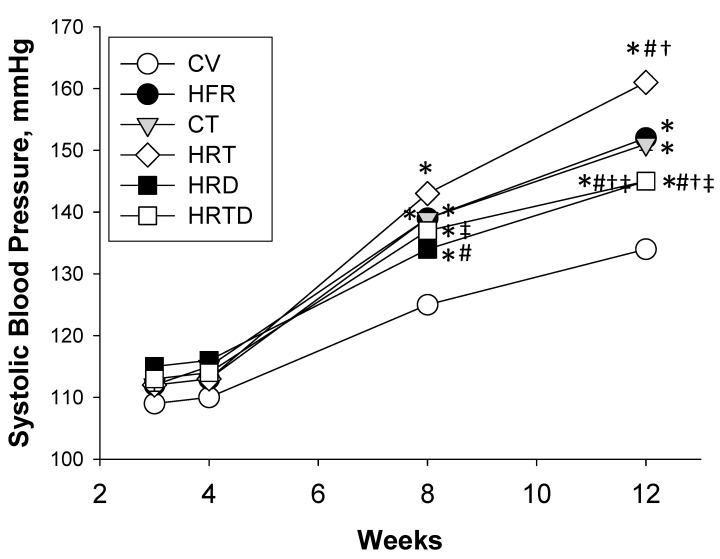
Effects of high-fructose intake, 2,3,7,8-tetrachlorodibenzo-p-dioxin (TCDD) exposure, and 3,3-dimethyl-1-butanol (DMB) on systolic blood pressures in offspring from 3 to 12 weeks of age. CV = control rats received regular diet and vehicle; HFR = rats received high-fructose diet; CT = rat received regular diet and TCDD exposure; HRT = rats received high-fructose diet and TCDD exposure; HRD = rats received high-fructose diet and DMB treatment; HRDT = rats received high-fructose diet, TCDD exposure, and DMB treatment. Data were analyzed by two-way ANOVA with post-hoc Tukey’s test. *n* = 8/group. * *p* < 0.05 vs. CV; # *p* < 0.05 vs. HFR; † *p* < 0.05 vs. CT; ‡ *p* < 0.05 vs. HRT.

Figure 2 shows that the HFR, CT, and HRT groups had a significantly higher systolic BP compared to the CV group from 8 to 12 weeks of age. In week 12, the elevated SBP was exacerbated more by TCDD exposure in the HRT group than among those in the CV, HFR, and CT groups. A significant reduction in SBP (~16 mmHg) was measured in the HRTD group versus the HRT group. Additionally, DMB treatment caused a reduction in the SBP (~7 mmHg) in the HRD group, compared to the HFR group. Our data indicated the synergistic interaction between HFR and TCDD influencing the elevation of the BP, which was attenuated by DMB treatment. The HFR (*p* = 0.04), and the CT group (*p* < 0.001) had a higher creatinine (Cr) level compared with the CV group, while the Cr levels in the HRT, HRD, and HRTD group were similar to the CV group.

### 2.2. TMA, TMAO, and DMA

We first determined the plasma levels of TMA, TMAO, and dimethylamine (DMA, the metabolite of TMAO and TMA) (Table 2). Maternal high-fructose intake significantly increased plasma TMA levels (HFR vs. CV group, *p* = 0.045), while TCDD exposure had no effect on the levels. The plasma TMAO level was higher in the HRD group compared to the HRT group (*p* = 0.027). The CT (*p* = 0.011), HRT (*p* = 0.001), and HRD group (*p* = 0.002) had lower plasma DMA levels compared with the HFR group. 

### 2.3. NO-Related Metabolites

As shown in Table 2, the plasma levels of asymmetric and symmetric dimethylarginine (ADMA and SDMA, endogenous inhibitors of nitric oxide synthase) were higher, but the L-arginine-to-ADMA ratios were lower in the HRF and CT groups compared to the controls (all *p* < 0.05). DMB treatment caused decreases in the ADMA (*p* = 0.003) and SDMA (*p* = 0.003) levels in the HRD group vs. the HFR group. Additionally, a high-fructose diet significantly reduced the L-arginine-to-ADMA ratio in the HFR group compared to the controls (*p* = 0.022). The decrease in this ratio was restored by DMB treatment in the HRD group. However, NO-related metabolites were comparable between the HRT and HRTD group.

### 2.4. Renin-Angiotensin System

**Figure 3:**
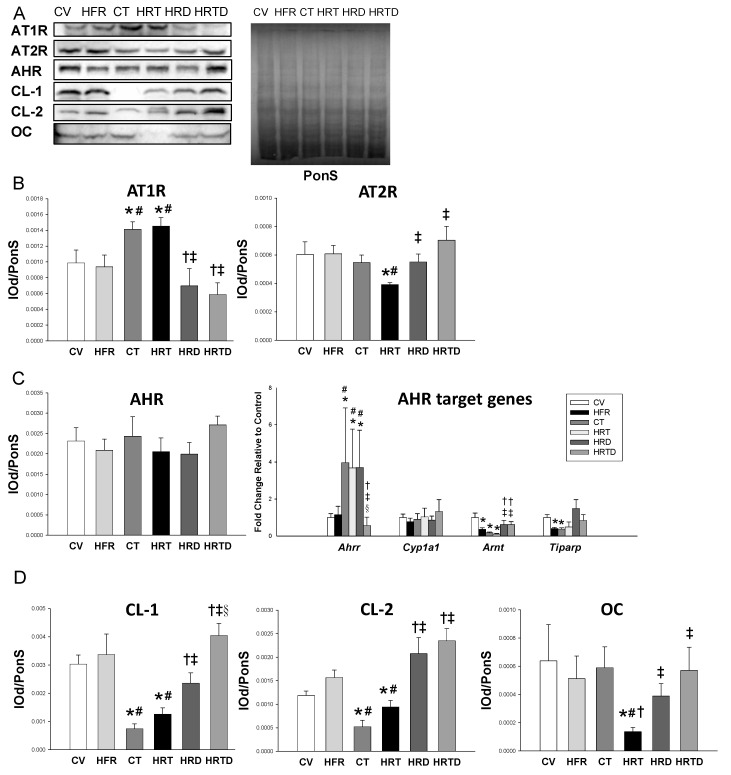
(**A**) Representative Western blots and Ponceau S red (PonS) staining showing angiotensin II type 1 receptor (AT1R; 43 kDa), angiotensin II type 2 receptor (AT2R; 50 kDa), aryl hydrocarbon receptor (AHR; 96 kDa), claudin-1 (CL-1, 23kDa), claudin-2 (CL-2, 25kDa), and occludin (OC; 65kDa) in offspring at 12 weeks of age. (**B**) Relative abundance of AT1R and AT2R in the kidneys. (**C**) The protein level of AHR and mRNA expression of AHR target genes, including *Ahrr, Cyp1a1, Arnt,* and *Tiparp* were analyzed by qPCR. (**D**) The relative abundance of CL-1, CL-2, and OC in the ileum. CV = control rat received regular diet and vehicle; HFR = rats received high-fructose diet; CT = rats received regular diet and TCDD exposure; HRT = rats received high-fructose diet and TCDD exposure; HRD = rats received high-fructose diet and DMB treatment; HRDT = rats received high-fructose diet, TCDD exposure, and DMB treatment. Data were analyzed by one-way ANOVA with post hoc Tukey’s test. *n* = 8/group. * *p* < 0.05 vs. CV; # *p* < 0.05 vs. HFR; † *p* < 0.05 vs. CT; ‡ *p* < 0.05 vs. HRT; § *p* < 0.05 vs. HRD.

In addition to NO, dysregulation of the RAS is a key mechanism involved in programmed hypertension. We analyzed protein levels of RAS components in offspring kidneys. We observed there was a significant increase in the protein level of angiotensin II type 1 receptor (AT1R) in the CT and HRT groups vs. controls, which DMB therapy prevented (Figure 3). Conversely, the renal protein level of angiotensin II type 2 receptor (AT2R) was lower in the HRT group compared to the controls. DMB therapy restored the decreased AT2R protein abundance in the HRT group (Figure 3B).

### 2.5. AHR Signaling Pathway

We next determined AHR and its target genes in offspring kidneys. Figure 3C shows that there was no difference in renal AHR protein level among the six groups. Renal mRNA expression of *Ahrr* was higher in the CT, HRT, and HRD groups compared to the controls (all *p* < 0.05). Additionally, TCDD caused a reduction in *Arnt* and *Tiparp* expression. We found that these gene changes in the AHR signaling pathway were recovered by DMB therapy (both *p* < 0.05).

### 2.6. Gut Permeability and Microbiota Composition

We examined the ileal level of tight junction proteins to determine gut permeability. Ileal clausin-1 and -2 expression significantly decreased in the CT and HRT groups’ kidneys, and was restored by DMB therapy (Figure 3D). The occludin protein level was decreased in the HRT group, and was restored by DMB treatment.

**Figure 4:**
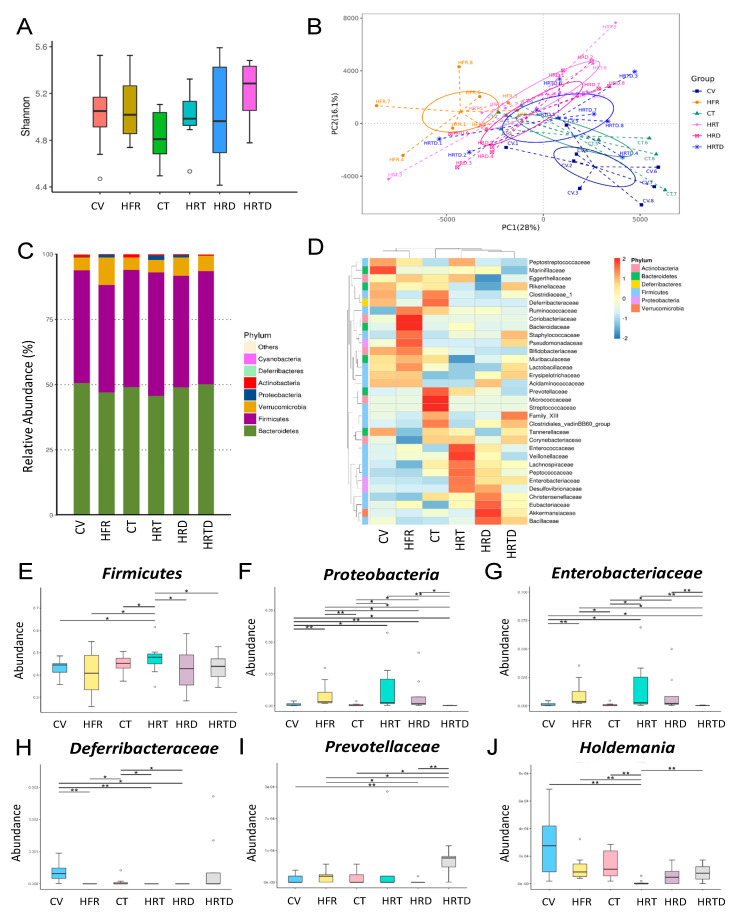
Effects of high-fructose intake, 2,3,7,8-tetrachlorodibenzo-p-dioxin (TCDD) exposure, and 3,3-dimethyl-1-butanol (DMB) on the gut microbiota in offspring at 3 weeks of age. (**A**) α-diversity was represented by the Shannon’s indexes. (**B**) β-diversity changes in gut microbiota across groups were shown by principal component analysis (PCA). (**C**) Relative abundance of the top 10 phyla of the gut microbiota among the six groups. (**D**) Heat map of the 16S rRNA gene sequencing analysis of the gut microbiome at the family level. The abundance of (**E**) phylum *Firmicutes*, (**F**) phylum *Proteobacteria*, (**G**) family *Enterobacteriaceae*, (**H**) family *Deferribacteraceae*, (**I**) family *Prevotellaceae*, and (**J**) genus *Holdemania* among the six groups. CV = control rat received regular diet and vehicle; HFR = rats received high-fructose diet; CT = rats received regular diet and TCDD exposure; HRT = rats received high-fructose diet and TCDD exposure; HRD = rats received high-fructose diet and DMB treatment; HRDT = rats received high-fructose diet, TCDD exposure, and DMB treatment. * *p* < 0.05. ** *p* < 0.01.

We further analyzed the composition of the gut microbiota in offspring at 3 and 12 weeks of age. Microbiome diversity is typically defined in terms of within (i.e., α-diversity) and between (i.e., β-diversity) microbial community diversities [17,18]. The Shannon diversity index is a commonly used α-diversity measure to determine how evenly the microbes are distributed in a community/sample [19]. We found that there was no significant difference in the Shannon index among the six groups at 3 weeks of age (Figure 4A). We used two different β-diversity analysis techniques, principal component analysis (PCA) and analysis of similarities (ANOSIM), to compare the bacterial community similarities [17,18]. PCA can reduce the dimensionality of microbiome data sets so that a summary of the beta diversity relationships can be visualized in two-dimensional scatterplots [18]. ANOSIM was used to compare the differences among the groups of samples [20]. The scatterplots of PCA showed that some (e.g., HRT vs. HRTD), but not all, groups were well-separated (Figure 4B), while ANOSIM analysis showed there to be a significant difference between the five groups (all *p* < 0.05), except between the HRD and HRT groups (*p* = 0.095). These findings suggest that most groups are distinct. At 3 weeks of age, we observed that the main phyla were *Bacteroidetes, Firmicutes, Verrucomicrobia, Proteobacteria,* and *Actinobacteria* (Figure 4C). The abundance of the phyla *Firmicutes* (*p* = 0.038) and *Proteobacteria* (*p* = 0.035) was increased in the HRT group, which DMB treatment prevented (*p* = 0.033 and *p* < 0.001, respectively) (Figure 4E,F). The *Firmicutes* to *Bacteroidetes* ratio was comparable between groups. At the family level, HFR, TCDD, and DMB resulted in differential changes in the abundance of gut microbes (Figure 4D). We observed that the abundance of the family *Enterobacteriaceae* (*p* = 0.014) was higher in the HRT group vs. the control CV group (Figure 4G), which DMB treatment prevented (*p* < 0.001). In contrast, the HRT group had a significantly decreased abundance of the family *Deferribacteraceae* (Figure 4H, *p* < 0.001). The abundance of the family *Prevotellaceae* was significantly increased in the HRTD group compared to other groups (Figure 4I). Additionally, we found that the abundance of the genus *Holdemania* was lower in the HRT group compared to the control (Figure 4J, *p* < 0.001), which was prevented by DMB treatment (*p* < 0.001).

**Figure 5:**
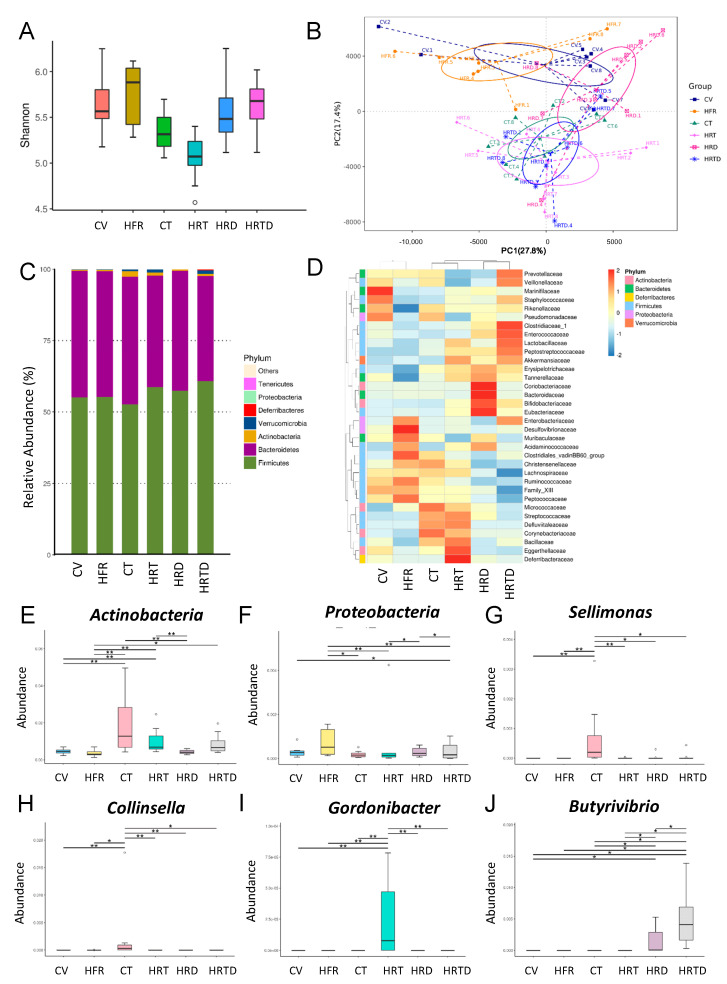
Effects of high-fructose intake, 2,3,7,8-tetrachlorodibenzo-p-dioxin (TCDD) exposure, and 3,3-Dimethyl-1-butanol (DMB) on the gut microbiota in offspring at 12 weeks of age. (**A**) α-diversity was represented by the Shannon’s indexes. (**B**) β-diversity changes in gut microbiota across groups were shown by principal component analysis (PCA). (**C**) Relative abundance of the top 10 phyla of the gut microbiota among the six groups. (**D**) Heat map of the 16S rRNA gene sequencing analysis of the gut microbiome at the family level. The abundance of (**E**) phylum *Actinobacteria*, (**F**) phylum *Proteobacteria*, (**G**) genus *Sellimonas*, (**H**) genus Collinsella, (**I**) genus *Gordonibacter*, and (**J**) genus *Butyrivibrio* among the six groups. CV = control rats received regular diet and vehicle; HFR = rats received high-fructose diet; CT = rats received regular diet and TCDD exposure; HRT = rats received high-fructose diet and TCDD exposure; HRD = rats received high-fructose diet and DMB treatment; HRDT = rats received high-fructose diet, TCDD exposure, and DMB treatment. * *p* < 0.05. ** *p* < 0.01.

As in week 3, the α-diversity was not different among the six groups in week 12 (Figure 5A). However, the PCA plots demonstrated that the composition of microbial communities of some groups (e.g., CV vs. HRTD) are well separated, while others overlap (e.g., CT vs. HRTD). ANOSIM showed that most groups were significantly different (all *p* < 0.05), except the difference between the HRT and HRTD groups did not reach significance (Figure 5B) (ANOSIM, *p* = 0.111). Figure 5C shows that the major phyla in week 12 were like those in week 3. At the family level, the effects of HFR, TCDD, and DMB on gut microbes are different between 3 and 12 weeks of age (Figure 5D). Compared to controls, the abundance of the phyla *Actinobacteria* (*p* = 0.007) and *Proteobacteria* (*p* = 0.031) was significantly increased in the HRT group and HRTD group, respectively (Figure 5E,F). We observed that TCDD exposure increased the abundance of the genera *Sellimonas* and *Collinsella* in the CT group vs. other groups (Figure 5G,H). Additionally, combined HFR plus TCDD exposure significantly increased the abundance of the genus *Gordonibacter* compared to other groups (Figure 5I). Moreover, the HRTD group had the highest abundance of genus *Butyrivibrio* among the six groups (Figure 5J).

## 3. Discussion

This is the first study to describe how DMB protects against TCDD plus HFR-induced hypertension, and makes special emphasis on the AHR signaling, gut microbiota, the RAS, and the NO pathway. The main findings of this research were: (1) TCDD exacerbates HFR-induced hypertension in male adult offspring, which can be attenuated by DMB therapy during gestation and lactation; (2) both HFR and TCDD exposure-induced programmed hypertension are associated with increased ADMA and SDMA, but a decreased L-arginine-to-ADMA ratio; (3) HFR plus TCDD-induced hypertension is associated with increased AT1R and decreased AT2R, which DMB prevents; (4) TCDD-induced elevation of BP is combined with increased AT1R, activation of AHR signaling, and increased gut permeability; (5) different maternal exposures induced gut microbiota dysbiosis and distinct enterotypes; and (6) DMB therapy affected several gut microbes related to TMA-TMAO metabolism, including the phyla *Firmicutes* and *Proteobacteria*, families *Enterobacteriaceae* and *Deferribacteraceae*, and genus *Holdemania*.

Our results reconfirm the results of previous studies demonstrating that excessive consumption of fructose or dioxin exposure by pregnant mother rats increases the risk of adult male offspring developing hypertension and kidney damage [3,8]. Most human exposure to dioxins is through food, mainly animal fats. Although animal fats and fructose are commonly consumed as part of human Western diets, no study to date has examined how early combined exposure to HFR and TCDD increases the risk of developing hypertension and kidney disease in later life. Since pregnant women are increasingly exposed to multiple adverse environmental insults and chemical hazards in modern society, our study casts new light on early microbiome-based metabolite treatments which could be used to prevent hypertension programmed by two-hit insults in later life. Our current study identified certain mechanisms underlying the protective effects of DMB against programmed hypertension, such as the increase in NO bioavailability, the balance of RAS, the abrogation of AHR activation, and the restoration of gut microbiota.

First, we observed that both HFR and TCDD affect the NO pathway. The index of NO bioavailability, the L-arginine-to-ADMA ratio, was recovered by DMB therapy in the HFD vs. HFR group. Additionally, DMB caused a reduction in the BP and was associated with a reduction in the NOS inhibitors ADMA and SDMA, which were induced by HFR intake. Our results suggest a link between the TMA-TMAO and ADMA-NO pathways, which concurs with previous studies showing their impacts in hypertension [12,21].

Second, our data showed that DMB therapy restored the combined HFR+TCDD-induced increased AT1R and decreased AT2R protein abundance in the HRTD group. It is well-established that AT2R appears to represent an endogenous counter-regulatory pathway within the RAS, the actions of which are in opposition to the vasoconstrictor Ang II/ACE/AT1R arm [22]. A previous study showed that TMAO is involved in the pathogenesis of angiotensin II-induced hypertension [23]. Although DMB protecting the HRTD group against hypertension is independent of TMA-TMAO formation, our data suggested that the protective mechanism of DMB against hypertension, at least in part, is related to the restoration of the RAS balance in the kidneys. Since DMB therapy affected several gut microbes related to TMA-TMAO metabolism, it is presumable that its beneficial effect on BP is related to the regulation of other microbiota-derived metabolites. Given that microbiota metabolite short chain fatty acids (SCFAs) could affect BP [11], and that DMB therapy was reported to regulate SCFAs [12], whether DMB therapy protected against hypertension is via SCFAs awaits further clarification.

Another beneficial effect of DMB therapy could be due to antagonizing AHR-mediated gene transcription. It has been reported that TCDD induces a high BP, and AHR target genes might be involved in this process [8,14]. The AHRR is an AHR target gene that competes with AHR to bind with ARNT [14]. Our results demonstrate that DMB restored TCDD-induced increased *Ahrr* but decreased *Arnt* mRNA expression in the kidneys of the offspring. Our data support the hypothesis that TCDD activates the AHR signaling pathway, which appears correlated with the rise in BP. Since the activation of the AHR/ARNT/CYP axis has been reported to induce vasoconstriction [24], this notion is supported by our findings. However, we observed that *cyp1a1* expression was not altered; other CYP enzymes await further evaluation. Although our data demonstrated no differences in AHR protein levels among the six groups, mRNA expressions of AHR target genes were changed in some groups. This discrepancy is due to that fact that protein abundance of a transcription factor does not necessarily result in its transcriptional activity. AHR can be sequestered away from nuclear DNA target genes when not actively transcribing target genes [25]. Thus, whether DMB differentially regulates the transcriptional activity of AHR resulting in different expression of AHR target gene warrants further investigation.

Moreover, data from the present study indicate that HFR, TCDD, and DMB are associated with distinct enterotypes, represented by β-diversity changes. In week 3, TCDD has little effect on the microbiota as compared with HFR. In the present study, the abundance of *Firmicutes* and *Proteobacteria*, the two main phyla in the TMA-producing community [26], were higher in the HRT group in week 3, while their increases were suppressed by DMB therapy. There was no difference in the *Firmicutes* to *Bacteroidetes* ratio among the six groups, despite this ratio being considered a microbial marker for hypertension [10]. According to our data, the HRT group showed an increase in the family *Enterobacteriaceae* but a decreased abundance of the family *Deferribacteraceae* and genus *Holdemania*. These changes were restored by DMB therapy. At the family level, *Enterobacteriaceae* made the greatest contribution to the conversion of TMAO to TMA [27], whereas *Deferribacteraceae* was involved in TMA production [28]. Overall, these observations indicate that certain bacteria populations involved in TMA metabolism were affected by DMB therapy at week 3. It is possible that the changes in specific microbial abundance contributed to the balance of the TMA-TMAO pathway, and the beneficial effects this had on hypertension in week 12. At 12 weeks of age, hypertension programmed by TCDD exposure was related to an increased abundance of the genus *Collinsella*, which was in line with the finding that *Collinsella* abundance is higher in atherosclerosis patients [29]. In contrast, the reduction in BP in the HRTD group was associated with a high abundance of the genus *Butyrivibrio*. Our finding ties in well with a previous report linking reduced *Butyrivibrio* abundance to hypertensive populations [30].

One limitation of this study is a lack of the control + DMB and TCDD + DMB group. One reason for this is because a previous study showed a good safety profile of DMB administration in controls [31]. Another reason is due to the fact that we mainly focused on studying the reprogramming effect of DMB on the two-hit model, instead of the one-hit model. Nevertheless, whether DMB administration in pregnancy and lactation might cause long-term effects in offspring prenatally exposed to TCDD or in normal controls remains to be clarified. Secondly, we only analyzed gut microbiota in offspring, but not in dams. Whether different insults experienced by mothers could regulate the gut microbiota in both dams and offspring, and whether maternal gut microbiota is related to offspring outcome, both require further evaluation. Thirdly, we are well aware that the mechanisms analyzed in the present study might not represent the whole picture of the protective role of DMB therapy. Since maternal HFR-induced hypertension is related to epigenetic regulation and nutrient-sensing signals [5,32], additional studies are needed to elucidate whether these mechanisms are also associated with the protective effects of DMB. Lastly, we mainly focused on the kidneys in the present study. Accordingly, we know very little about what role other organs play in the BP-lowering effect of DMB.

## 4. Materials and Methods

### 4.1. Animals and Experimental Design

Virgin Sprague–Dawley (SD) rats (BioLASCO Taiwan Co., Ltd., Taipei, Taiwan) were housed in a facility accredited by the Association for Assessment and Accreditation of Laboratory Animal Care International. The rats were housed under 12 h light/12 h dark conditions. This study was conducted under the Institutional Animal Care and Use Committee protocol approved (Permit number: 2019011001; approval date: 31 January 2019) by the Kaohsiung Chang Gung Memorial Hospital, and complies with the National Institutes of Health’s Guide for the Care and Use of Laboratory Animals. Mating was achieved by placing one female and one male in a cage overnight, and successful mating was confirmed by observation of a vaginal plug.

To construct a maternal HFR model, pregnant dams fed with high-fructose (60% fructose) diet throughout pregnancy and lactation [5]. Additionally, pregnant rats exposed to an oral dose of TCDD at 200 ng/kg body weight (Sigma-Aldrich, St. Louis, MO, USA) or an oral dose of corn oil vehicle at 4 mL/kg BW on gestational day 14 and 21, and days 7 and 14 after birth, to provide both in utero and lactation exposure. Because TCDD has a long half-life of ~3 weeks in rats, TCDD was used at weekly doses based on previous studies [8,33]. One group of mother rats exposed to HFR or HFR+TCDD received 1% DMB in the drinking water during the pregnancy and lactation period. The dose of DMB used here was based on findings of previous studies [12,16]. To standardize the received quantity of milk and maternal pup care, the litters were culled to a total of eight pups after birth. Cardiovascular events occur at an earlier age in males than females [34]. We, therefore, only selected male offspring at random from each litter for subsequent experiments. Male offspring were assigned to six groups (*n* = 8 for each group): regular diet (CV), high-fructose diet (HFR), regular diet+TCDD exposure (CT), HFR+TCDD exposure (HRT), high-fructose diet+DMB treatment (HRD), and HFR+TCDD+DMB treatment (HRTD).

At 3 weeks of age, male offspring were weaned and placed onto the regular chow ad libitum from weaning to 12 weeks of age. We used the BP-2000 Blood Pressure Analysis system (BP-2000, Visitech Systems, Inc., Apex, NC, USA) to measure the BP in conscious rats at intervals of four weeks, beginning from 4 weeks and through to 12 weeks of age. This device used the tail-cuff method. To ensure accuracy and reproducibility, the rats were acclimated to restraint and tail-cuff inflation for 1 week prior to the measurement [5].

Fresh feces samples were collected at 3 and 12 weeks of age, frozen, and placed in a −80 °C freezer for further analysis. At 12 weeks of age, all rats were killed. Blood samples were collected in heparinized tubes. The kidneys were harvested and placed in a −80 °C freezer until analysis. Plasma creatinine levels were analyzed by HPLC according to our previous protocol [12].

### 4.2. Liquid Chromatography–Mass Spectrometry (LC–MS/MS) Analysis

Plasma levels of TMA, TMAO, and DMA were determined by LC–MS/MS analysis. The detection was performed by an Agilent 6410 Series Triple Quadrupole mass spectrometer (Agilent Technologies, Wilmington, DE, USA) with an electrospray ionization source, as described previously [12]. The mass spectrometric determination was carried out using multiple reaction monitoring mode. The precursor to product ion transitions of *m*/*z* 60.1→44.1, *m*/*z* 76.1→58.1, and *m*/*z* 46.1→30 were selected for the quantification of TMA, TMAO and DMA, respectively. Diethylamine was used as an internal standard.

### 4.3. High Performance Liquid Chromatography (HPLC)

Plasma levels of NO-related metabolites, including L-citrulline (the precursor of L-arginine), L-arginine (substrate for NO synthesis), SDMA, and SDMA were measured using HPLC (HP series 1100; Agilent Technologies Inc., Santa Clara, CA, USA). O-phthalaldehyde/3-mercaptopropionic acid (OPA/3MPA) was used as the derivative reagent [5]. Homoarginine (Sigma) was used as the internal standard.

### 4.4. Metagenomics Analysis of Gut Microbiota

Frozen fecal samples were analyzed with metagenomics focused on the V3–V4 of the 16S DNA gene. As described previously [12], all polymerase chain-reaction amplicons were mixed and sent to the Biotools Co., Ltd. (Taipei, Taiwan). Libraries were sequenced with Illumina MiSeq platform (Illumina, San Diego, CA, USA). Illumina sequence data were processed using QIIME version 1.9.1. A median of 99,198 raw sequencing reads and 80,622 effective tag sequences per sample was obtained, respectively. The sequences were clustered into operational taxonomic units (OTUs) at 97% similarity using the USEARCH algorithm. The phylogenetic structure analysis was constructed with Fast-Tree. We compared the patterns of α- and β- diversity for microbial communities [17,18]. The α-diversity analysis, accounting for both the abundance and evenness of the taxa present, was calculated using the Shannon index in QIIME 1.9.1 [19]. We evaluated the β-diversity changes in gut microbiota across groups using PCA and ANOSIM [20].

### 4.5. Quantitative Real-Time PCR Analysis

RNA was extracted from the kidney cortex [8]. Two-step quantitative real-time PCR (qPCR) was conducted using Quantitect SYBR Green PCR Reagents (Qiagen, Valencia, CA, USA) on an iCycler iQ Multi-Color Real-Time PCR Detection System (Bio-Rad, Hercules, CA, USA). Four AHR target genes were analyzed, including *Ahrr* (encoding for the aryl hydrocarbon receptor repressor), *Cyp1a1* (Cytochrome P450 CYP1A1), *Arnt* (encoding for the aryl hydrocarbon receptor nuclear translocator), and *Tiparp* (encoding for TCDD-inducible poly-ADP-ribose polymerase). *Rn18s* was used as a reference in all analyses. The sequences of the primers used in this study are provided in Table 3. All samples were run in duplicate. The comparative threshold cycle (CT) method was used for the relative quantification of gene expression. The averaged CT was subtracted from the corresponding averaged *Rn18s* value for each sample, resulting in the Δ*C*_T_. ΔΔ*C*_T_ was achieved by subtracting the average control Δ*C*_T_ value from the average experimental Δ*C*_T_. The fold change was 2^−ΔΔ*C*T^.

### 4.6. Western Blot

The Western blot analysis was performed on the kidney cortex or ileal homogenate, as previously described [5]. We used 10–15% polyacrylamide gels and separated by electrophoresis (200 V, 90 min), then transferred onto polyvinylidene difluoride membranes. Following transfer, the membrane was stained with Ponceau S red (PonS) stain solution (Sigma-Aldrich, St. Louis, MO, USA) to verify equal loading. We analyzed the renal protein abundance of components in the RAS, including AT1R and AT2R. Additionally, we determined the expressions of AHR in the kidney, as well as in the tight junction proteins (occludin, claudin-1, and -2) in the ileal segment. A list of antibodies used for Western blotting is shown in Table 4. The Western blots were visualized using a SuperSignal West Pico reagent (Pierce; Rockford, IL, USA). Densitometry analysis was provided for each band, and was quantified as the integrated optical density (IOD) normalized to PonS staining.

### 4.7. Statistics

Data are expressed as the mean ± standard error of the mean. A *p*-value less than 0.05 is considered statistically significant for all tests. Two-way repeated-measures ANOVA and Tukey’s post hoc tests were used for BP analysis. Statistical analysis was undertaken using one-way ANOVAs with post hoc Tukey’s test for multiple comparisons. All analyses were performed using the Statistical Package for the Social Sciences (SPSS) 15.0 statistics software (SPSS Inc, Chicago, IL, USA).

## 5. Conclusions

In conclusion, the addition of TCDD exposure exacerbates hypertension programmed by maternal HFR exposure in adult male offspring. There are several protective mechanisms by which maternal DMB therapy moderates hypertension programmed by combined HFR+TCDD exposure, such as increasing NO bioavailability, balancing the RAS, antagonizing AHR signaling, and restoring the gut microbiota. Our findings cast new light on early interventions targeting microbiome-dependent metabolite TMA, which may aid in developing an ideal strategy to protect pregnant women and their children against hypertension programmed by excessive fructose consumption and dioxin exposure.

## Figures and Tables

**Table 1:** Morphological and biochemical values.

Groups	CV	HFR	CT	HRT	HRD	HRTD
Mortality	0%	0%	0%	0%	0%	0%
Body weight (g)	380 ± 9	366 ± 6	383 ± 17	390 ± 9	404 ± 13 ^#^	373 ± 7
LKW (g)	1.68 ± 0.05	1.59 ± 0.04	1.73 ± 0.08	1.79 ± 0.06 ^#^	1.69 ± 0.03	1.65 ± 0.04
LKW/BW (g/g)	0.44 ± 0.01	0.43 ± 0.01	0.45 ± 0.01	0.46 ± 0.1	0.42 ± 0.01 ^†,‡^	0.44 ± 0.01
SBP (mmHg)	134 ± 0	152 ± 1 ^*^	151 ± 1 ^*^	161 ± 1^*,#,†^	145 ± 1 ^*,#,†,‡^	145 ± 1 ^*,#,†,‡^
Creatinine (μM)	18.2 ± 1.9	23.3 ± 1.2 ^*^	30.5 ± 1.1 ^*,#^	21.2 ± 1.4 ^#,†^	19.4 ± 1.2 ^#,†^	22.7 ± 2.4 ^†^

CV = control rat received regular diet and vehicle; HFR = rats received high-fructose diet; CT = rat received regular diet and TCDD exposure; HRT = rats received high-fructose diet and TCDD exposure; HRD = rats received high-fructose diet and DMB treatment; HRDT = rats received high-fructose diet, TCDD exposure, and DMB treatment; BW= body weight; LKW = left kidney weight; SBP = Systolic blood pressure; *n* = 8/group. ^*^ *p* < 0.05 vs. CV; ^#^ *p* < 0.05 vs. HFR; ^†^ *p* < 0.05 vs. CT; ^‡^ *p* <0.05 vs. HRT.

**Table 2:** Plasma levels of methylamines and NO-related metabolites at 12 weeks of age.

Groups	CV	HFR	CT	HRT	HRD	HRTD
Methylamines						
TMA (ng/mL)	174 ± 13	209 ± 6 ^*^	200 ± 12	210 ± 13	172 ± 15	212 ± 17
TMAO (ng/mL)	252 ± 19	234 ± 17	244 ± 13	219 ± 14	262 ± 10 ^‡^	247 ± 24
DMA (ng/mL)	96 ± 5	107 ± 4	92 ± 4 ^#^	88 ± 3 ^#^	87 ± 3 ^#^	114 ± 10 ^‡,§^
NO-related metabolites						
Citrulline (μM)	62.7 ± 4.5	80.4 ± 8.4	65.3 ± 6	61.4 ± 6.7	44.8 ± 6.7 ^#^	50.1 ± 3.8 ^#^
Arginine (μM)	195 ± 14	214 ± 22	161 ± 16	172 ± 16	137 ± 13 ^*,#^	211 ± 19 ^§^
ADMA (μM)	1.5 ± 0.12	2.6 ± 0.25 ^*^	3.1 ± 0.2 ^*^	2 ± 0.25 ^†^	1.3 ± 0.21 ^#,†,‡^	2.5 ± 0.18^*,§^
SDMA (μM)	0.6 ± 0.09	1.9 ± 0.26 ^*^	1.4 ± 0.29 ^*^	1.4 ± 0.28 ^*^	0.7 ± 0.17 ^#,‡^	2.3 ± 0.45^*,§^
Arginine-to-ADMA ratio	137 ± 17	85 ± 7 ^*^	51 ± 5 ^*,#^	93 ± 14 ^†^	147 ± 43	88 ± 8 ^*,†^

CV = control rat received regular diet and vehicle; HFR = rats received high-fructose diet; CT = rat received regular diet and TCDD exposure; HRT = rats received high-fructose diet and TCDD exposure; HRD = rats received high-fructose diet and DMB treatment; HRDT = rats received high-fructose diet, TCDD exposure, and DMB treatment; TMA = trimethylamine; TMAO = trimethylamine N-oxide; DMA = dimethylamine; ADMA = asymmetric dimethylarginine; SDMA = symmetric dimethylarginine; *n* = 8/group. ^*^ *p*< 0.05 vs. CV; ^#^ *p* < 0.05 vs. HFR; ^†^ *p* < 0.05 vs. CT; ^‡^ *p* < 0.05 vs. HRT; ^§^ *p* < 0.05 vs. HRD.

**Table 3:** Quantitative real-time polymerase chain reaction primer sequences.

Gene	Forward (5′–3′)	Reverse (5′–3′)
*Ahrr*	cagcaacatggcttctttca	tgaagcactgcattccagac
*Cyp1a1*	gcactctggacaaacacctg	atatccaccttctcgcctgg
*Arnt*	gtctccctcccagatgatga	gctggtagccaacagtagcc
*Tiparp*	gttgagggccaattaccaga	gctcctggcacataatccat
*Rn18s*	gccgcggtaattccagctcca	cccgcccgctcccaagatc

*Ahrr* = Aryl hydrocarbon receptor repressor, *Cyp1a1* = Cytochrome P450 CYP 1A1, *Arnt* = Aryl hydrocarbon receptor nuclear translocator, *Tiparp* = TCDD-inducible poly-ADP-ribose polymerase, *Rn18s* = 18S ribosomal RNA (r18S).

**Table 4:** Antibodies used for Western blotting.

Antibody	Host	Source	Dilution
AT1R	Rabbit	Millipore	1:500
AT2R	Rabbit	Santa Cruz Biotechnology	1:250
AHR	Rabbit	Novus Biologicals	1:1000
CL-1	Rabbit	Abcam	1:500
CL-2	Rabbit	Thermo Fisher Scientific	1:500
OC	Rabbit	Thermo Fisher Scientific	1:500

AT1R = angiotensin II type 1 receptor; AT2R = angiotensin II type 2 receptor; AHR = aryl hydrocarbon receptor; CL-1 = claudin-1; CL-2 = claudin-2; OC = occludin.

## Abbreviations

ADMA	Asymmetric dimethylarginine
AHR	Aryl hydrocarbon receptor
ANOSIM	Analysis of Similarities
AT1R	Angiotensin II type 1 receptor
AT2R	Angiotensin II type 2 receptor
CL-1	Claudin-1
CL-2	Claudin-2
DOHaD	Developmental origins of health and disease
DMA	Dimethylamine
DMB	3,3-Dimethyl-1-butanol
FMO	Flavin-containing monooxygenase
OC	Occludin
PCA	Principal component analysis
PonS	Ponceau S red
RAS	Renin-angiotensin system
SD	Sprague–Dawley
SDMA	Symmetric dimethylarginine
TCDD	2,3,7,8-tetrachlorodibenzo-p-dioxin
TMA	Trimethylamine
TMAO	Trimethylamine N-oxide
